# Role of MyD88 in IL-1β and Ethanol Modulation of GABAergic Transmission in the Central Amygdala

**DOI:** 10.3390/brainsci9120361

**Published:** 2019-12-07

**Authors:** Michal Bajo, Reesha R. Patel, David M. Hedges, Florence P. Varodayan, Roman Vlkolinsky, Tony D. Davis, Michael D. Burkart, Yuri A. Blednov, Marisa Roberto

**Affiliations:** 1 Department of Molecular Medicine, The Scripps Research Institute, La Jolla, CA 92037, USAdmhedges@gmail.com (D.M.H.); varodaya@scripps.edu (F.P.V.); vlkolins@scripps.edu (R.V.); mroberto@scripps.edu (M.R.); 2 Department of Chemistry & Biochemistry, University of California, San Diego, La Jolla, CA 92093, USA; tdd005@ucsd.edu (T.D.D.); mburkart@ucsd.edu (M.D.B.); 3 Waggoner Center for Alcohol and Addiction Research, The University of Texas at Austin, Austin, TX 78712, USA; yablednov@austin.utexas.edu

**Keywords:** interleukin-1, IL-1R1, neuroimmune, alcohol, *Myd88* knockout, sIPSC, GABA

## Abstract

Myeloid differentiation primary response protein (MyD88) is a critical neuroimmune adaptor protein in TLR (Toll-like receptor) and IL-1R (Interleukin-1 receptor) signaling complexes. These two pro-inflammatory families play an important role in the neurobiology of alcohol use disorder, specifically MyD88 regulates ethanol drinking, ethanol-induced sedation, and ethanol-induced deficits in motor coordination. In this study, we examined the role of MyD88 in mediating the effects of IL-1β and ethanol on GABAergic transmission in the central amygdala (CeA) of male mice using whole-cell patch-clamp recordings in combination with pharmacological (AS-1, a mimetic that prevents MyD88 recruitment by IL-1R) and genetic (*Myd88* knockout mice) approaches. We demonstrate through both approaches that IL-1β and ethanol’s modulatory effects at CeA GABA synapses are not dependent on MyD88. *Myd88* knockout potentiated IL-1β’s actions in reducing postsynaptic GABA_A_ receptor function. Pharmacological inhibition of MyD88 modulates IL-1β’s action at CeA GABA synapses similar to *Myd88* knockout mice. Additionally, ethanol-induced CeA GABA release was greater in *Myd88* knockout mice compared to wildtype controls. Thus, MyD88 is not essential to IL-1β or ethanol regulation of CeA GABA synapses but plays a role in modulating the magnitude of their effects, which may be a potential mechanism by which it regulates ethanol-related behaviors.

## 1. Introduction

Acute and chronic ethanol exposure induce an innate immune response in the central nervous system (CNS) of humans, and this effect is replicated in preclinical models of alcohol use disorder (AUD) [1,2,3,4]. Most notably, genomic studies in mice [5], rats [6], and humans [3,7,8] have identified several members of the Toll-like receptors (TLR) and interleukin-1/interleukin18 (IL-1/IL-18) receptor pathways in AUD [9,10]. Moreover, pharmacological [11,12,13], viral [14,15], or transgenic [2,9,11,16,17,18,19,20] manipulation of the innate immune system leads to alterations of the ethanol-related behaviors in rodents. Collectively, this growing literature on innate immune responses critically contributing to the neurobiology of AUD [21,22,23] suggests that these pathways have high potential as targets for the development of new AUD therapeutic strategies [16,24,25,26].

Initiation of the innate immune response can be mediated by TLR and IL-1 receptor families, which use a common adaptor protein—myeloid differentiation primary response protein (MyD88) [27,28]. After TLR/IL-1 receptor activation, MyD88 is recruited to the receptor’s intracellular domain where it interacts with interleukin receptor-associated kinase (IRAK) 4, which subsequently recruits IRAK1, IRAK2, and IRAK3 [29,30]. The MyD88/IRAK protein complex then activates various downstream intracellular signaling pathways, depending on the cell type, ligands and activated receptors present, and cellular location of the signal initiation [28], ultimately leading to the production of inflammatory mediators (e.g., TNF, IL-1, IL-6, CCL4) as part of the innate immune response [30,31].

A role for MyD88 in several ethanol-related behaviors was recently reported. Specifically, male *Myd88* knockout mice display increased ethanol drinking compared to wildtype mice [17]. In addition, MyD88 is a critical mediator of the sedative and intoxicating effects of ethanol and GABA receptor-acting sedatives (gaboxadol, diazepam, and pentobarbital) [18,19,20], suggesting that MyD88 plays a regulatory role on GABAergic transmission in the brain. Given the role of MyD88 in ethanol-related behaviors, a mechanistic understanding of MyD88 actions in mediating these behaviors is needed.

In this study, we tested whether MyD88 mediates the effects of IL-1β and ethanol on GABA transmission in the central nucleus of the amygdala (CeA). CeA GABAergic transmission plays a critical role in mediating ethanol and anxiety-like behaviors [32], and we have previously identified modulatory roles for several innate immune elements [including LPS (a TLR4 agonist, [33]), IL-1β, the IL-1R1 ligand, and IL-1ra (an endogenous IL-1R1 antagonist [34,35])] at mouse CeA GABAergic synapses. Moreover, acute ethanol potentiates CeA GABAergic transmission [36,37,38,39]. For these experiments, we conducted whole-cell patch-clamp recording of the spontaneous inhibitory postsynaptic currents (sIPSCs) in CeA neurons following genetic knockout or pharmacological inhibition of MyD88 signaling. Our results suggest that although MyD88 is not necessary for IL-1β and ethanol’s regulation of GABAergic signaling, it critically modulates the overall magnitude of IL-1β and ethanol’s effect at these synapses.

## 2. Materials and Methods

### 2.1. Animals

We used adult male *Myd88* knockout (KO) mice (provided by Dr. Blednov from the University of Texas at Austin, Austin, TX; originated from Jackson Laboratories; *n* = 19; age: 28.7 ± 1.4 weeks old; weight: 30.53 ± 0.65 g) and C57BL/6J wildtype (WT) control male mice (Jackson Laboratories; *n* = 17; age: 25.8 ± 1.6 weeks old; weight: 31.47 ± 0.97 g). The mice were housed in a temperature- and humidity-controlled room on a 12 h light cycle (lights on from 06:00–18:00), and food and water were available ad libitum. In our care of the mice, we followed the National Institutes of Health Guide for the Care and Use of Laboratory Animals and the Institutional Animal Care and Use Committee policies of The Scripps Research Institute (animal protocol #09-0006-4 pproved on 28 September 2017). 

### 2.2. Slice Preparation

We anesthetized each mouse with 3%–5% isoflurane and isolated the brain in ice-cold gassed (95% O_2_ and 5% CO_2_) aCSF (composition in mM: NaCl, 130; KCl, 3.5; NaH_2_PO_4_, 1.25; MgSO_4_•7H_2_O, 1.5; CaCl_2_, 2.0; NaHCO_3_, 24; glucose, 10). Then, we transferred the brain into ice-cold high-sucrose cutting solution (in mM: 206 sucrose; 2.5 KCl; 0.5 CaCl_2_; 7MgCl_2_; 1.2 NaH_2_PO_4_; 26 NaHCO_3_; 5 glucose; 5 HEPES) and sectioned 300 µm coronal slices containing the CeA using a Vibratome 1200S (Leica Biosystems, Buffalo Grove, IL, Wetzlar, Germany). CeA slices recovered in oxygenated aCSF at 37 °C for 30 min and were then incubated at room temperature for a minimum of 30 min.

### 2.3. Whole-Cell Patch-Clamp Recording

We used infrared/DIC visualization [40], followed by digitization and image enhancement via an upright, fixed-stage Olympus microscope (BX51WI and BX50WI, Olympus Scientific Solutions America, Waltham, MA, USA) to identify CeA neurons. We performed whole-cell patch-clamp electrophysiology in voltage-clamp mode and recorded pharmacologically-isolated spontaneous inhibitory postsynaptic currents mediated by GABA_A_ receptors (sIPSCs) from neurons in the medial subdivision of the CeA, using bath application of the glutamatergic receptor blockers (20 μM DNQX and 30 μM DL-AP5) and GABA_B_ receptor blockers (1 μM CGP 55845A). Borosilicate glass micropipettes were pulled to a pipette resistance of 3–6 MΩ and were filled with an internal solution containing (in mM): 135 KCl; 10 HEPES; 2 MgCl_2_; 0.5 EGTA; 5 ATP; and 1 GTP (pH 7.3–7.4, osmolarity 295–315 mOsm). We held neurons at −60 mV and recorded sIPSCs in a gap-free mode at room temperature using Axon digitizers 1440 or 1550, Multiclamp amplifiers 700B and pClamp 10.2 software (Molecular Devices, Sunnyvale, CA, USA). Series resistance was not compensated, and access resistance was <20 MΩ with <20% change during the duration of the recording. The tested compounds—ethanol (44 and 100 mM), recombinant mouse IL-1β (50 ng/mL), and AS-1 (50 µM)—were applied directly to the bath in known concentrations. sIPSC measurements were taken before (baseline = 3 min preceding drug application) and during drug superfusion (7–15 min after drug application). For the recordings of the basal GABA sIPSCs, we used all the WT (*n* = 17) and KO (*n* = 19) mice. For the rest of our experimental conditions, we recorded from a minimum of 3–6 mice per group.

### 2.4. Data Analysis and Statistics

We analyzed whole-cell recordings with MiniAnalysis 5.1 software (Synaptosoft, Leonia, NJ, USA), and performed all statistical analysis with GraphPad Prism 8.0 (GraphPad Software, San Diego, CA, USA). Because of cell-to-cell variation in baseline electrophysiological properties, we normalized the effects of tested drugs to each neuron’s own baseline before group analysis. Experimental groups were divided on a cell-by-cell basis using the functional response into 3 groups: (1) a clear increase (>115%), (2) a clear decrease (<115%), (3) or no change over normalized baseline values [34,35,41]. The values are presented as means ± SEM, and we accepted statistical significance at the *p* < 0.05 level using one-sample- and unpaired *t*-tests.

### 2.5. Drugs

We purchased CGP 55845A, DNQX, and DL-AP5 from Tocris Biosciences (Ellisville, MI, USA) and recombinant mouse IL-1β from BioLegend (San Diego, CA, USA). We obtained ethanol from Remet (La Mirada, CA, USA). Hydrocinnamoyl-L-valyl-pyrrolidine (AS-1)—a low-molecular-weight MyD88 mimetic—was synthesized as previously described [42]. 

## 3. Results

### 3.1. Basal GABAergic Transmission is Similar in the CeA of Myd88 KO and WT Mice 

**Figure 1:**
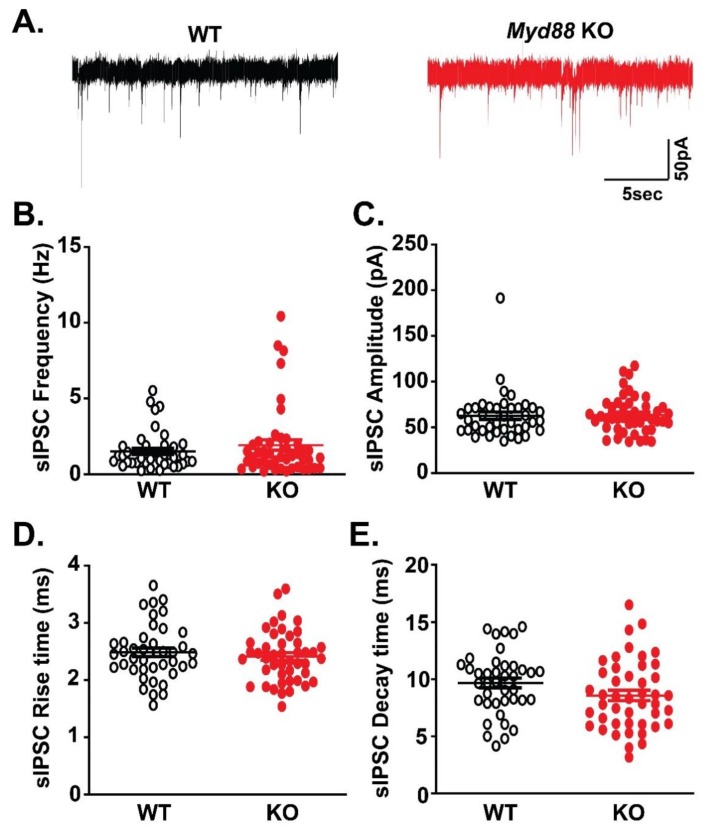
Basal spontaneous GABAergic transmission is similar in the CeA of *Myd88* KO and wildtype (WT) mice. (**A**) Representative traces of sIPSCs from WT and *Myd88* KO mice. (**B**–**E**) There were no significant differences in the basal sIPSC frequencies (**B**), amplitudes (**C**), rise times (**D**), and decay times (**E**) of CeA neurons from WT (*n* = 41 neurons) and *Myd88* KO (*n* = 45 neurons) mice. The scattergrams represent values for each cell. Statistical significance was calculated by an unpaired *t*-test, and significance was set at *p* < 0.05.

We recorded spontaneous inhibitory postsynaptic currents mediated by GABA_A_ receptors (sIPSCs) in the medial subdivision of the CeA of WT (41 neurons) and *Myd88* KO mice (45 neurons). There were no significant genotypic differences in the mean sIPSC frequency (Figure 1A,B; WT: 1.49 ± 0.20 Hz vs. KO: 1.94 ± 0.35 Hz), amplitude (Figure 1C; WT: 62.37 ± 4.03 pA vs. KO: 63.74 ± 3.04 pA), rise time (Figure 1D; WT: 2.47 ± 0.08 ms vs. KO: 2.41 ± 0.07 ms), and decay time (Figure 1E; WT: 9.62 ± 0.42 ms vs. KO: 8.57 ± 0.45 ms). In general, decreased sIPSC frequency can be associated with a lower neurotransmitter release probability, while changes in sIPSC amplitude and kinetics can be linked to altered postsynaptic receptor composition/expression/function [43]. Therefore, MyD88 does not regulate basal GABAergic transmission in the CeA.

### 3.2. MyD88 Modulates IL-1β’s Actions at CeA GABA Synapses

**Figure 2:**
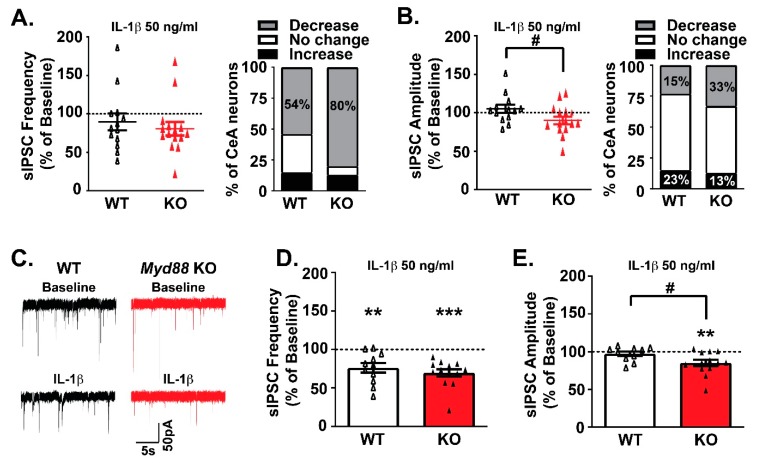
MyD88 deletion dampens IL-1β’s effects on postsynaptic GABA_A_ receptor function. (**A**–**B**) IL-1β had dual effects on the sIPSC frequencies (**A**) and amplitudes (**B**) in both WT and KO mice. The scattergrams on the left show the normalized effects of IL-1β (50 ng/mL) in individual cells (WT: *n* = 13 cells; KO: *n* = 15 cells), and the right panels show the percentage of the CeA neurons responding to IL-1β with an increase, no change or decrease in the sIPSC frequencies and amplitudes. (**C**) Representative traces of CeA neurons responding to IL-1β with decreased sIPSC frequencies and amplitudes. (**D**–**E**) While there were no differences in the predominant effect of IL-1β on the mean sIPSC frequency in WT (*n* = 11 cells) and KO (*n* = 13 cells) mice (**D**), *Myd88* KO mice showed an IL-1β-induced decrease in the sIPSC amplitude (**E**). The statistical significance for the IL-1β effects was calculated by one-sample *t*-test (** *p* < 0.01, and *** *p* < 0.001), and for the comparison of the IL-1β effects between the WT and KO mice, an unpaired *t*-test was used (# *p* < 0.05).

We then assessed the role of MyD88 in IL-1β’s effects on CeA GABA transmission using *Myd88* knockout mice. In agreement with our previous findings [34,41], bath application of IL-1β (50 ng/mL) had dual effects on CeA sIPSC frequency and amplitude in WT mice (Figure 2A,B). Similar dual effects on GABA release and postsynaptic GABA_A_ receptor function were observed in the *Myd88* KO mice. Notably, IL-1β decreased sIPSC frequency in the majority of CeA neurons across both genotypes (WT: 54% of cells and KO mice: 80% of cells; Figure 2A), and the extent of the effects are comparable. Specifically, IL-1β reduced GABA release to a similar extent in WT (76.02% ± 6.15% of baseline, *n* = 11; one-sample *t*-test, *p* < 0.01) and KO CeA neurons (69.33% ± 4.88% of baseline, *n* = 13; one-sample *t*-test, *p* < 0.0001; Figure 2C,D). Thus, MyD88 is not necessary for IL-1β’s presynaptic effect at GABA synapses.

The distribution of IL-1β-induced sIPSC amplitude responses was also similar across genotypes, but the overall effect of IL-1β was significantly different (*t*-test, *p* < 0.05; Figure 2B). Two CeA neurons from each genotype displayed a similar increase in sIPSC amplitude. However, when grouping the remaining CeA neurons for IL-1β responsivity [41], IL-1β induced a significant decrease in the sIPSC amplitude of *Myd88* KO mice (85.01% ± 4.21% of baseline, *n* = 13; one-sample *t*-test, *p* < 0.01), but not WT mice (97.30% ± 3.06% of baseline, *n* = 10) (Figure 2C,E). There were no significant IL-1β-induced differences in the sIPSC kinetics across genotypes (data not shown). Thus, although MyD88 is not critical for IL-1β’s regulation of GABA_A_ receptor function, MyD88 modulates the magnitude of these synaptic responses in CeA neurons.

### 3.3. Pharmacological Inhibition of MyD88 Modulates IL-1β’s Action at CeA GABA Synapses Similar to Myd88 KO Mice

**Figure 3:**
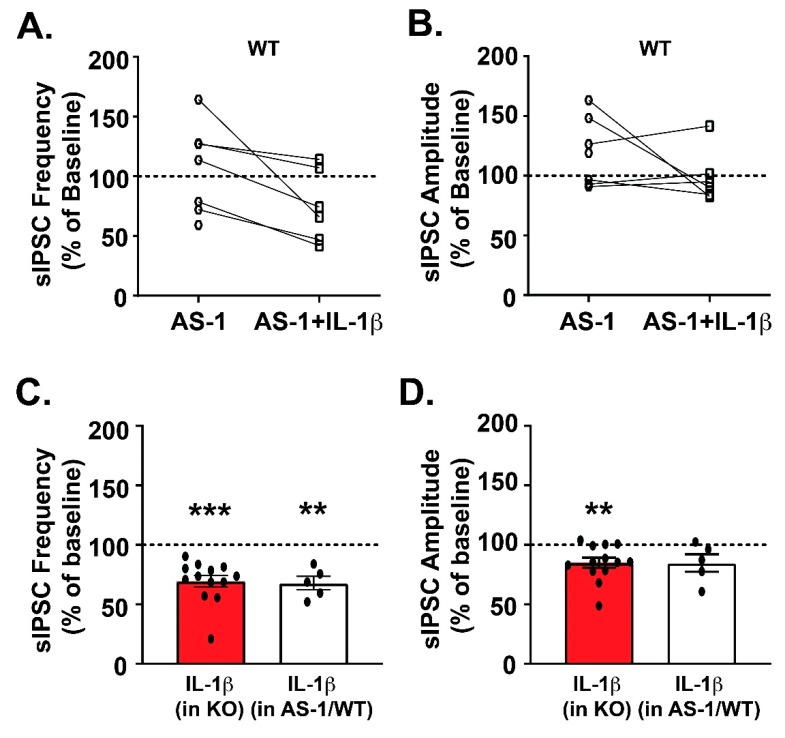
The effects of the MyD88 mimetic (AS-1) in WT mice. (**A**–**B**) The scattergrams represent the responses of individual CeA cells to the acute application of the AS-1 (50 mM) and subsequent co-application of IL-1β, where panel **A** shows the effects of AS-1 on the sIPSC frequencies (WT: *n* = 7 cells), and panel **B** shows its effects on the sIPSC amplitude. (**C**–**D**). In five out of six WT neurons pretreated with AS-1, there was an IL-1β-induced decrease in the mean sIPSC frequency, and the magnitude of this effect was not significantly different from the IL-1β-induced decrease in the *Myd88* KO mice (data from Figure 2D). (**D**) IL-1β in the presence of AS-1 had no significant effects on the mean sIPSC amplitudes of WT cells, and the extent of this effect was not significantly different from the IL-1β’s effects in the *Myd88* KO mice (data from Figure 2E). The statistical significance for the AS-1 and IL-1β effects was calculated by one-sample *t*-test (** *p* < 0.01 and *** *p* < 0.0001), and for the comparison of the IL-1β’s effects between the WT, following AS-1 pretreatment, and KO mice, an unpaired *t*-test was used.

We next used a mimetic of MyD88 (AS-1) to pharmacologically interfere with IL-1R1/MyD88 signaling to parallel our *Myd88* gene deletion studies. AS-1 binds to the IL-1R1′s TIR domain to prevent MyD88 recruitment specifically to the IL-1R1 signaling complex, without compromising other pathways that employ MyD88 signaling [44,45]. Thus, AS-1 pretreatment of WT CeA slices allows us to mimic the deletion of the MyD88 (*Myd88* KO) without compromising TLR-MyD88 signaling. We first assessed the baseline effect of the pharmacological interference and found that AS-1 alone (50 µM) had dual effects on sIPSC frequency; three out of seven neurons responded with an increase (139.6% ± 12.34% of baseline; one-sample *t*-test, *p* > 0.05), one out of seven neurons had no effect (113.51% of baseline), and three out of seven responded with a decrease (70.03% ± 5.7% of baseline; one-sample *t*-test, *p* < 0.05) (Figure 3A). AS-1′s effect on postsynaptic receptor function were more consistent with an increase in sIPSC amplitude observed in four out of seven CeA neurons (139.3% ± 10.10% of baseline; one-sample *t*-test, *p* < 0.05), and no effect (93.51% ± 1.66% of baseline) in the rest of the cells (Figure 3B). AS-1’s effect on the kinetics were mixed (data not shown), with the majority of cells showing increased sIPSC rise time (115.0% ± 5.752% of baseline, *n* = 7; one-sample *t*-test, *p* < 0.05) and decay time (122.9% ± 5.48% of baseline, *n* = 7; one-sample *t*-test, *p* < 0.01).

After 15 min of AS-1 application, we co-applied IL-1β (50 ng/mL) in a subset of WT cells. IL-1β decreased the mean sIPSC frequency (compared to the AS-1 baseline, which corresponds to the last 3 min of AS-1 application before the co-application of IL-1β) in 83% of neurons (five out of six cells, 67.96% ± 5.67% of the AS-1 baseline; one-sample *t*-test, *p* < 0.01; Figure 3C) and increased it in 17% of cells (one out of six cells). These IL-1β effects are similar to those observed in *Myd88* KO mice (Figure 2D).

Likewise, in the presence of AS-1, there was a trend toward significance for IL-1β to decrease the mean sIPSC amplitude in the majority of CeA neurons (84.81% ± 7.35% of AS-1 baseline, *n* = 5 out of 6 cells; one-sample *t*-test, *p* = 0.10; Figure 3D). Co-application of AS-1 and IL-1β did not induce significant changes in the kinetics (data not shown). These IL-1β-induced changes on GABA release and postsynaptic GABA_A_ receptor function following AS-1 pretreatment resemble those observed in the *Myd88* KO mice, both in terms of the distribution of individual cell responses and the overall effects, providing further evidence that MyD88 is not necessary for IL-1β’s effect on CeA GABA transmission but modulates it.

### 3.4. MyD88 Modulates Ethanol’s Facilitation of GABA Release in the CeA

**Figure 4:**
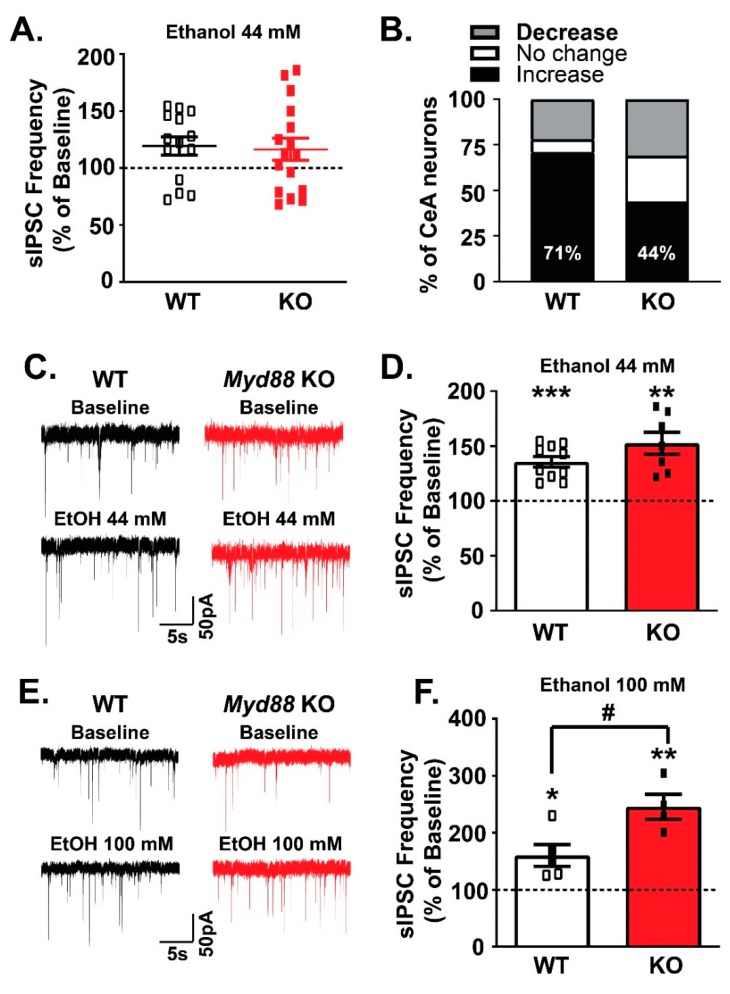
Facilitation of the presynaptic GABA release by 100 mM ethanol is more robust in *Myd88* KO than in the WT mice. (**A**,**B**). Ethanol (44 mM) facilitated presynaptic GABA release across both genotypes, as represented by the increase in the mean sIPSC frequencies in 10 out of 14 neurons from WT and 7 out of 16 cells from KO mice. (**C**) Representative recordings of the CeA neurons responding to 44 mM ethanol with the increase in the sIPSC frequencies. (**D**) There was no significant difference in the magnitude of the ethanol-induced potentiation of the GABA release between the WT and KO mice. (**E**) Representative traces of the CeA neurons responsive to 100 mM ethanol. (**F**) The magnitude of the 100 mM ethanol-induced potentiation of the GABA release was significantly stronger in the KO mice (*n* = 4 out of 5 cells) compared to the WT mice (*n* = 5 out of 5 cells) mice. The statistical significance for the ethanol effects was calculated by one-sample *t*-test (* *p* < 0.05, ** *p* < 0.01, and *** *p* < 0.001), and an unpaired *t*-test (# *p* < 0.05) was used for the comparison of the magnitude of the ethanol effects between the WT and KO mice.

To examine the potential role of MyD88 in acute ethanol-induced facilitation of CeA GABAergic transmission, we compared the effects of 44 and 100 mM [33] of ethanol on sIPSCs in WT and KO mice. While a majority (71% of cells) of WT neurons responded to acute application of 44 mM ethanol [34,38,39,41,46,47] with a significant increase in the mean sIPSC frequency, only half of cells (44% of cells) from the KO mice did (Figure 4A,B). Examining only this predominant effect of ethanol’s facilitation of GABA release, there was no difference (unpaired *t*-test, *p* > 0.05) across genotypes (WT: 135.6% ± 4.83% of baseline, *n* = 10, one sample *t*-test, *p* < 0.001; vs. KO: 152.6% ± 9.96% of baseline, *n* = 7; one-sample *t*-test, *p* < 0.01; Figure 4C,D). However, a higher concentration of ethanol (100 mM) induced significantly (unpaired *t*-test, *p* < 0.05) stronger potentiation of sIPSC frequency in CeA neurons from KO mice (245% ± 21.9% of baseline, *n* = 4 out of 5 cells; one-sample *t*-test, *p* < 0.01) compared to WT mice (160.4% ± 19.12%, *n* = 5 out of 5 cells; one-sample *t*-test, *p* < 0.05) (Figure 4E,F).

Although acute 44 mM ethanol did not significantly alter sIPSC amplitudes (WT and KO: 109% ± 5.09% and 105.7% ± 3.66% of baseline, respectively), it significantly increased sIPSC decay times in both groups (WT: 115.2% ± 3.747%, one-sample *t*-test, *p* < 0.05; KO: 112.8% ± 3.807%, one-sample *t*-test, *p* < 0.05) and increased the rise time only in WT mice (105.2% ± 2.153%, one-sample *t*-test, *p* < 0.05; data not shown). 100 mM ethanol did not significantly alter sIPSC amplitudes (WT and KO: 124.0% ± 18.22% and 118.2% ± 15.08% of baseline, respectively) or rise times between the WT and KO mice, but increased the sIPSC decay time in WT mice (114% ± 2.887% of baseline one-sample *t*-test, *p* < 0.01) but not in KO mice (101.3% ± 12.74% of baseline one-sample *t*-test, *p* = 0.924; data not shown). 

## 4. Discussion

In this study, we examined the role of MyD88 in mediating the effects of IL-1β and ethanol on GABAergic transmission in the central amygdala. We used both transgenic (*Myd88* knockout) and pharmacological (a mimetic preventing recruitment of MyD88 to IL-1R1) approaches. Through both approaches, we found that MyD88 is not necessary for the effects of either IL-1β or ethanol on CeA GABAergic transmission. However, *Myd88* knockout potentiated IL-1β’s actions to reduce postsynaptic GABA_A_ receptor function. Similarly, high-dose (100 mM) ethanol-induced GABA release was greater in the CeA of *Myd88* knock out mice compared to wild type mice. Thus, while MyD88 is not essential to IL-1β or ethanol regulation of CeA GABA synapses, it plays a role in modulating the magnitude of their effects. Because we performed all our experiments using male mice, we cannot rule out potential sex differences in the role of MyD88 on IL-1β and ethanol effects on the CeA GABA transmission.

MyD88 plays a central role in mediating the immune response after activation of IL-1R1 [27], but the activation of IL-1R1 also induces MyD88-independent signaling [42]. The MyD88-independent pathway has been shown to mediate fast, transcription-independent signaling, including activation of Akt via binding of the PI3-kinase p85 subunit to IL-1R1 without MyD88 association [42]. Our findings suggest that MyD88-independent signaling is also involved in IL-1β regulation of GABA transmission in the CeA. Previously, we found that the application of the endogenous IL-1ra antagonist or its deletion (*Il1rna* KO mice) decreased or increased baseline CeA GABAergic transmission, respectively [34,35], indicating a constitutive role for the IL-1R1 at these synapses. Here, we report no significant differences in the basal GABA transmission between the *Myd88* KO and WT mice, suggesting that IL-1R1’s regulation of the basal CeA GABAergic transmission may also be, in part, mediated by MyD88-independent signaling pathways.

We observed dual pre- and post-synaptic effects of IL-1β on GABA transmission in both WT and *Myd88* KO mice, which are in accordance with our previous findings [34,35,41]. While genotype differences in IL-1β’s effects were not observed, we found significant differences in the magnitude of the overall postsynaptic effect of IL-1β between WT and KO. The deletion of MyD88 resulted in a stronger reduction of the amplitude of spontaneous GABAergic currents in the CeA neurons, suggesting that MyD88 may play a modulatory role and tune the GABA response to IL-1β, particularly attenuating IL-1β-induced postsynaptic inhibition of the GABA response. In general, the sIPSC amplitude may be affected by several factors, including the number of GABA_A_ receptors at the synapse, receptor desensitization, receptor subunit composition, phosphorylation of GABA_A_ receptors, and the spatiotemporal profile of the GABA concentration [48].

One of the caveats of using a general knockout approach is the potential development of compensatory changes. Therefore, we also used a pharmacological approach based on the MyD88 mimetic AS-1, which is specific for IL-1R1-induced activation of the MyD88 signaling [44,45]. Thus, AS-1 allowed us to pharmacologically inhibit IL-1R1-induced MyD88-dependent signaling without affecting TLR-MyD88 signaling. AS-1 disrupts the recruitment of MyD88 with IL-1R1 at the TIR domain, where it imitates a tri-peptide sequence [(F/Y)-(V/L/I)-(P/G)] of the TIR domain BB-loop [44], and subsequently, attenuates IL-1-induced activation of IRAK-1 and p38 MAPK signaling [44,45]. We observed that inhibition of the IL-1β-induced MyD88 signaling by AS-1 had effects on the baseline GABAergic transmission in the CeA neurons in WT mice. Notably, AS-1 had dual effects on the presynaptic GABA release, represented by the changes in the sIPSC frequency; while postsynaptically, AS-1 significantly increased the kinetics of the GABA transmission in the WT. Thus, our data also support a partial role of the IL-1R1 in the regulation of the basal CeA GABAergic transmission [34,35]. Interestingly, activation of the IL-1R1 with exogenous IL-1β in the presence of AS-1 resulted in decreased presynaptic GABA in the WT animals, further supporting our findings that MyD88-independent signaling mediates IL-1R1-induced inhibitory effects on the CeA GABA transmission. Of note, we cannot rule out a possibility that compensatory mechanisms in the MyD88-downstream signaling pathways might affect some of our results. Further studies will be needed to tease out specific downstream signaling pathways mediating the modulatory role of MyD88 in the effects of IL-1β.

We have previously shown that acute application of ethanol facilitates presynaptic GABA release in rodent [34,35,36,37,38,39] and non-human primate CeA neurons [49]. In the present study, the deletion of MyD88 did not prevent ethanol-induced potentiation of the GABA release, indicating that MyD88 is not critically involved in the facilitatory effects of ethanol on CeA GABA transmission. Although we did not observe a significant difference in the number of ethanol-responsive neurons in the KO mice compared to WT, the number of CeA neurons responding to moderate concentration of ethanol (44 mM) dropped to about 50% of the recorded cells. The high ethanol concentration (100 mM) reduced the heterogeneity in the ethanol responses as all cells from WT and the vast majority of cells from KO mice responded to the high dose of ethanol with increased presynaptic GABA release. Notably, the facilitation of the GABA release was significantly stronger in the *Myd88* KO than in WT mice, which indicates a potential modulatory role of MyD88 in ethanol’s effects on CeA GABA release. Thus, similar to IL-1β effects, MyD88 seems to attenuate the presynaptic effects of the high ethanol concentration.

Based on behavioral studies showing a regulatory role of MyD88 on GABAergic transmission [18], here, we assessed the role of MyD88 in the GABAergic effects of IL-1β and ethanol in the CeA. Our findings indicate that IL-1β and ethanol regulation of GABAergic signaling does not require MyD88, but MyD88 can modulate (attenuate) the GABAergic effects of IL-1β and high ethanol concentrations in the CeA. The CeA is sensitive to ethanol, and the overactivation of its GABA signaling is considered a hallmark of the transition to alcohol dependence. Our current findings complement our previous studies on the regulation of GABA transmission in the CeA by IL-1 and CD14/TLR4, both of which signal through MyD88. We have shown that IL-1 signaling is involved in basal GABAergic transmission and that IL-1β and CD14/TLR4 interact with the acute ethanol effects on GABA in the CeA [33,34,35]. We have also shown that ethanol dependence induces dysregulation of the IL-1 system, represented by increased IL-1β expression in the CeA, without altering its neuromodulatory role on synaptic transmission [41]. Overall, this set of studies indicates that specific innate immune elements modulate basal GABAergic transmission in the CeA differently. In addition, the chronic ethanol-induced neuroimmune response in the CeA may contribute to the neuroadaptive changes in the CeA that mediate ethanol-dependence-related behaviors [50]. At the behavioral level, the roles of specific innate immune elements of TLR4 and IL-1 system in the ethanol-related behaviors vary. While TLR4, IL-1R1, IL-1ra, and MyD88 play an important role in acute ethanol-induced sedation and motor impairment, CD14 does not [12,18,51]. Similar specificity is observed with ethanol drinking, where CD14, IL-1ra, and MyD88 (only in males) play significant roles [17,51], while TLR4, IL-1R1, and MyD88 (in females) are not involved in the regulation of ethanol consumption [12,16,17]. This recurrent specificity in the roles of individual components of the innate immunity in ethanol’s cellular and behavioral effects indicate that any new AUD therapeutical strategy targeting the innate immune system must be tailored to specific aspects of the disease. Thus, understanding the ethanol-induced immune response and specific downstream signaling at the cellular, brain region, and behavioral levels is a critical first step for the identification of new therapeutic strategies.
